# Procedural volume and outcomes in patients undergoing VA-ECMO support

**DOI:** 10.1186/s13054-020-03016-z

**Published:** 2020-06-05

**Authors:** Peter Moritz Becher, Alina Goßling, Benedikt Schrage, Raphael Twerenbold, Nina Fluschnik, Moritz Seiffert, Alexander M. Bernhardt, Hermann Reichenspurner, Stefan Blankenberg, Dirk Westermann

**Affiliations:** 1 Department of Cardiology, University Heart and Vascular Center Hamburg, Martinistrasse 52, 20246 Hamburg, Germany; 2 German Center for Cardiovascular Research (DZHK), Partner Site Hamburg/Kiel/Lübeck, Hamburg, Germany; 3 Cardiovascular Research Institute Basel (CRIB) and Department of Cardiology, University Hospital Basel, University of Basel, Basel, Switzerland; 4 Department of Cardiovascular Surgery, University Heart and Vascular Center Hamburg, Hamburg, Germany

**Keywords:** Venoarterial extracorporeal membrane oxygenation, VA-ECMO, Procedure volume, Outcomes, Complications

## Abstract

**Background:**

Venoarterial extracorporeal membrane oxygenation (VA-ECMO) is increasingly used in patients with critical cardiopulmonary failure. To investigate the association between hospital VA-ECMO procedure volume and outcomes in a large, nationwide registry.

**Methods:**

By using administrative data from the German Federal Health Monitoring System, we analyzed all VA-ECMO procedures performed in Germany from 2013 to 2016 regarding the association of procedural volumes with outcomes and complications.

**Results:**

During the study period, 10,207 VA-ECMO procedures were performed; mean age was 61 years, 43.4% had prior CPR, and 71.2% were male patients. Acute coronary syndrome was the primary diagnosis for VA-ECMO implantation (*n* = 6202, 60.8%). The majority of implantations (*n* = 5421) were performed at hospitals in the lowest volume category (≤ 50 implantations per year).

There was a significant association between annualized volume of VA-ECMO procedures and 30-day in-hospital mortality for centers with lower vs. higher volume per year. Multivariable logistic regression showed an increased 30-day in-hospital mortality at hospitals with the lowest volume category (adjusted odds ratio 1.13, 95% confidence interval [CI] 1.01–1.27, *p* = 0.034).

Similarly, higher likelihood for complications was observed at hospitals with lower vs. higher annual VA-ECMO volume (adjusted odds ratio 1.46, 95% CI 1.29–1.66, *p* = 0.001).

**Conclusions:**

In this analysis of more than 10,000 VA-ECMO procedures for cardiogenic shock, the majority of implantations were performed at hospitals with the lowest annual volume. Thirty-day in-hospital mortality and likelihood for complications were higher at hospitals with the lowest annual VA-ECMO volume.

## Introduction

Venoarterial extracorporeal membrane oxygenation (VA-ECMO) is increasingly used for treatment of patients with critical cardiopulmonary failure and as a rescue therapy to stabilize critically ill patients with circulatory compromise [1, 2]. In the last decade, VA-ECMO therapy has seen a rapid increase in the western world, although in-hospital mortality rates remained at a high level [3, 4]. We have recently reported that utilization of VA-ECMO for cardiopulmonary support increased by more than 30-fold in Germany between 2007 and 2015, while in-hospital mortality remained unchanged around 60% [5].

As a result of the rapid increase in VA-ECMO implantations, the number of implanting centers has also increased. To what extent an increase of the VA-ECMO implanting hospitals and their procedure volume of VA-ECMO implantations has a role in terms of outcome remains unclear. Several studies have shown that higher hospital procedure volume is associated with improved outcomes in interventional procedures such as transcatheter aortic-valve replacement [6], percutaneous mitral valve repair [7], percutaneous coronary intervention [8], and coronary artery bypass grafting [9]. It is speculated that this observation is the expression of a learning curve for interventional procedures. Whether this might be translated to VA-ECMO implantation is currently unknown.

Importantly, not only mortality, but also incident complication would be an outcome of interest, as 50% of all VA-ECMO patients experience therapy-limiting complications such as major bleeding, stroke, limb and abdominal ischemia, thrombosis, and infection [10–12]. Nevertheless, whether treatment of these complications in higher volume centers affects outcome is unknown.

The aim of this study was to investigate the association between hospital VA-ECMO procedure volume and outcomes in a large, nationwide registry. Findings regarding volume-mortality association could be of importance to inform the complex decision-making processes when considering initiation of VA-ECMO therapy for cardiogenic shock.

## Methods

### Data source and study population

In Germany, administrative data on characteristics and outcomes of all in-patients treated in German hospitals are obligatorily and routinely collected and reported to the Federal Statistical Office. Completely anonymized patient-level data are centrally stored and managed by the Research Data Center of the Federal Bureau of Statistics (Wiesbaden, Germany).

For the present analyses, all cases treated with VA-ECMO between 2013 and 2016 were identified and selected by the primary operation and procedural (OPS) code “VA-ECMO” (OPS code 8852.3) during index-hospitalization. The study population was divided into three categories of annualized hospital procedure volume (≤ 50 procedures, > 50–100 procedures, and >  100 procedures).

**Fig. 1:**
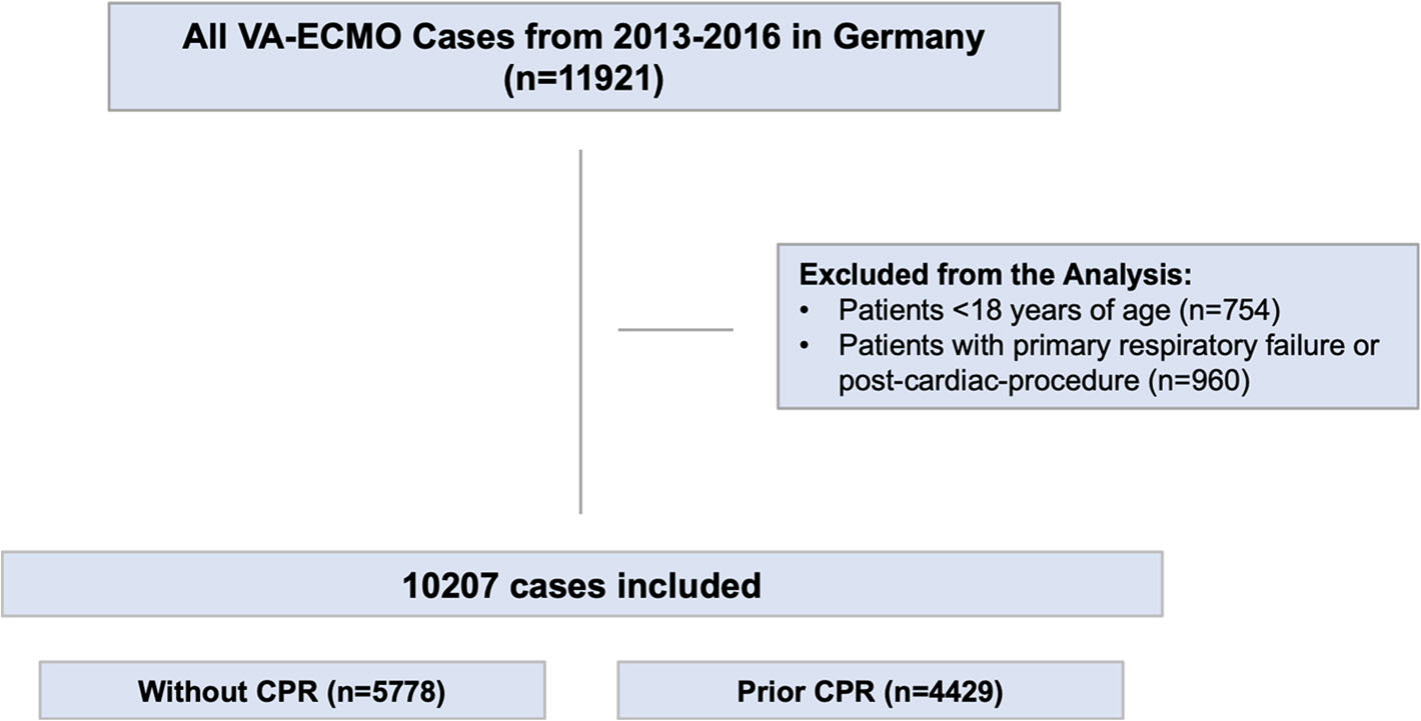
Flow chart of the study population

To obtain data on coexisting conditions, outcomes, and complications, we used the available German modification of international statistical classification of diseases and related health problems (ICD-10-GM) and OPS codes (Table I, II, and III in the online-only Data Supplement). Individuals younger than 18 years of age and patients with primary respiratory failure or post-cardiac-procedure as leading indication for VA-ECMO were excluded (Fig. 1 and Table II in the online-only Data Supplement). For subgroup analysis, patients with prior cardiopulmonary resuscitation (CPR) were identified and selected by OPS code 8871. The investigators did not have direct access to the raw data but were allowed to submit statistical scripts to the Research Data Center which performed the analyses and returned summarized statistical reports.

### Indications for VA-ECMO utilization

To characterize the primary indication for VA-ECMO utilization during index-hospitalization, patients were allocated into one of three mutually exclusive categories based on suggestions of recent consensus guidelines [13]: complications post heart transplantation (abbreviated as heart transplantation), acute coronary syndrome, and acute heart failure (Table II in the online-only Data Supplement). The assignment of indication categories was hierarchical if more than one of the three indications was formally present. For example, a patient was only considered for the acute heart failure category if he did not undergo heart transplantation and did not suffer from acute coronary syndrome in parallel. To avoid categorization based on diagnoses that developed as complications of VA-ECMO placement, only diagnosis codes present at the time of admission were considered.

### Statistical analysis

All individuals were divided into three hospital procedure volume groups. Binary variables were shown as absolute numbers and percentages, whereas continuous variables were shown as mean ± standard deviation (SD). For between-group comparisons, a one-way ANOVA test was used for continuous variables and the *χ*^2^ test for binary variables. For pairwise comparisons, adjusted *p* values by Holm were computed [14]. *χ*^2^ test was also used for comparisons between the overall cohort and the subset cohort of prior CPR patients. A multivariable logistic regression model was fitted to investigate the association of hospital procedure volume with 30-day in-hospital mortality, adjusted for age, sex, prior CPR, for the indication categories (post heart transplantation (HTX), acute coronary syndrome (ACS) and acute heart failure (AHF)), duration of VA-ECMO support, and complications.

Additionally, a multinomial logistic regression model was fitted to investigate the association between hospital procedure volume and complications. This model was adjusted for age, sex, prior CPR, for the indication categories (HTX, ACS, AHF), duration of VA-ECMO support, and time to 30-day in-hospital mortality.

Complications considered in both logistic regression analysis (either as dependent or as independent variable) were bleeding, stroke, abdominal ischemia, and limb ischemia during the index hospital stay (variable definitions are provided in Table III in the online-only Data Supplement).

All statistical methods were written in IBM SPSS Statistics 26 and were performed at the Federal Bureau of Statistics in Germany.

## Results

### Study population

Between 2013 and 2016, 10,207 patients ≥ 18 years of age received VA-ECMO for temporary circulatory support in Germany. Mean age of the overall cohort was 61 years (SD 17 years), 71.2% were male patients, and prior CPR was observed in 43.4%. In this cohort, the leading indication for VA-ECMO was ACS in 60.8%, AHF in 37.5%, and HTX in 1.8% (Table 1). Crude 30-day in-hospital mortality was 60.6% (Table 2).

**Table 1:** Baseline characteristics of the overall cohort according to annualized hospital volume of VA-ECMO procedures

	Baseline characteristics				*P v*alue
	Overall (***n*** = 10,207)	≤ 50 procedures/Yr (***n*** = 5421)	> 50–100 procedures/Yr (***n*** = 2799)	> 100 procedures/Yr (***n*** = 1987)	
**Demographics,*n*(%)**					
Mean age, Yr	61 ± 17	62 ± 19	61 ± 14	61 ± 14	< 0.001
Male gender	7272 (71.2)	3879 (71.5)	1998 (71.4)	1395 (70.2)	0.515
CPR	4429 (43.4)	2637 (48.6)	1104 (39.4)	668 (34.6)	< 0.001
**Baseline comorbidities,*n*(%)**					
AF	4031 (39.5)	2849 (36.2)	1171 (41.8)	893 (44.9)	< 0.001
CAD	6659 (65.2)	3713 (68.4)	1741 (62.2)	1205 (60.6)	< 0.001
CHF	6803 (66.7)	3570 (65.8)	1789 (63.9)	1444 (72.7)	< 0.001
CKD	2441 (23.9)	1382 (25.4)	655 (23.4)	404 (20.3)	< 0.001
COLD	514 (5.0)	331 (6.1)	136 (4.9)	47 (2.4)	< 0.001
PH	1783 (17.5)	761 (14.0)	541 (19.3)	481 (24.2)	< 0.001
HTN	4821 (47.2)	2542 (46.8)	1373 (49.1)	906 (45.6)	0.047
HLD	2531 (24.8)	1351 (24.9)	698 (24.9)	482 (24.3)	0.825
Diabetes	2571 (25.2)	1383 (25.5)	669 (23.9)	519 (26.1)	0.159
Cancer	278 (2.7)	169 (3.1)	79 (2.8)	30 (1.5)	0.001
Liver disease	507 (5.0)	238 (4.3)	147 (5.3)	122 (6.1)	0.006
Mean VA-ECMO duration in h (mdn)	116 (72)	98 (72)	128 (120)	150 (120)	0.001
**Indication category for VA-ECMO support,*n*(%)**					
HTX	179 (1.8)	22 (0.4)	75 (2.7)	82 (4.1)	< 0.001
ACS	6202 (60.8)	3528 (65.0)	1583 (56.6)	1091 (54.9)	< 0.001
AHF	3826 (37.5)	1871 (34.5)	1141 (40.8)	814 (41.0)	< 0.001

*Abbreviations: ACS* acute coronary syndrome, *AF* atrial fibrillation, *AHF* acute heart failure, *CAD* coronary artery disease, *CHF* congestive heart failure, *CPR* cardiopulmonary resuscitation, *CKD* chronic kidney disease, *COLD* chronic obstructive lung disease, *PH* pulmonary hypertension, *h* hours, *HTN* hypertension, *HLD* hyperlipidemia, *mdn* median, *n* number, *Yr* year

**Table 2:** Thirty-day in-hospital mortality and complications of the overall cohort according to annualized hospital volume of VA-ECMO procedures

	30-day in-hospital mortality and complications				
	Overall (***n*** = 10,207)	≤ 50 procedures/Yr (***n*** = 5421)	> 50–100 procedures/Yr (***n*** = 2799)	> 100 procedures/Yr (***n*** = 1987)	***P v***alue
**30-day in-hospital mortality,*n*(%)**					
30-day in-hospital mortality	6190 (60.6)	3550 (65.0)	1578 (56.4)	1062 (53.4)	< 0.001
**Complications,*n*(%)**					
Bleeding	2044 (20.0)	798 (14.7)	620 (22.2)	626 (31.5)	< 0.001
Stroke	105 (1.0)	47 (0.8)	38 (1.4)	20 (1.0)	0.112
Abdominal ischemia	711 (7.0)	277 (5.1)	215 (7.7)	219 (11.1)	< 0.001
Limb ischemia	751 (7.4)	325 (5.9)	244 (8.7)	182 (9.2)	< 0.001

*Abbreviations: n* number, *Yr* year

Subsequent analyses were performed for 4429 patients (43.4%) with prior CPR. These patients had a mean age of 61 years (SD 20 years), and 72.0% were male patients. The leading indication for the VA-ECMO was ACS in 66.3%, AHF in 32.9%, and HTX in 0.9% patients with prior CPR (Table 3). Crude 30-day in-hospital mortality of patients with prior CPR was 60.6% (Table 4). Detailed reports of the baseline characteristics and comorbidities depending on VA-ECMO procedural volume of the overall cohort and patients with prior CPR are shown in Tables 1 and 3.

**Table 3:** Baseline characteristics of all patients with prior CPR according to annualized hospital volume of VA-ECMO procedures

	Baseline characteristics				
	Overall (***n*** = 4429)	≤ 50 procedures/Yr (***n*** = 2637)	> 50–100 procedures/Yr (***n*** = 1104)	> 100 procedures/Yr (***n*** = 688)	***P v***alue
**Demographics,*n*(%)**					
Mean age, Yr	61 ± 20	61 ± 23	61 ± 14	61 ± 14	0.173
Male gender	3189 (72.0)	1915 (72.6)	801 (72.6)	473 (68.8)	0.118
**Baseline comorbidities,*n*(%)**					
AF	1510 (34.1)	815 (30.9)	399 (36.1)	296 (43.0)	< 0.001
CAD	3102 (70.0)	1875 (71.1)	766 (69.4)	461 (67.0)	0.097
CHF	2780 (62.8)	1629 (61.8)	670 (60.7)	481 (69.9)	< 0.001
CKD	974 (22.0)	607 (23.0)	238 (21.6)	129 (18.8)	0.051
COLD	187 (4.2)	106 (4.0)	50 (4.5)	31 (4.5)	0.004
PH	574 (13.0)	292 (11.1)	154 (13.9)	128 (18.6)	< 0.001
HTN	2032 (45.9)	1172 (44.4)	534 (48.4)	326 (47.4)	0.062
HLD	1016 (22.9)	582 (22.1)	264 (23.9)	170 (24.7)	0.230
Diabetes	1115 (25.2)	664 (25.2)	276 (25.0)	175 (25.4)	0.979
Cancer	130 (2.9)	80 (3.0)	37 (3.4)	13 (1.9)	0.183
Liver disease	187 (4.2)	106 (4.0)	50 (4.5)	31 (4.5)	0.719
Mean VA-ECMO duration in h (mdn)	98 (72)	85 (24)	111 (72)	128 (72)	< 0.001
**Indication category for VA-ECMO support,*n*(%)**					
HTX	39 (0.9)	6 (0.2)	13 (1.2)	20 (2.9)	< 0.001
ACS	2935 (66.3)	1797 (68.1)	717 (64.9)	421 (61.2)	< 0.001
AHF	1455 (32.9)	834 (31.6)	374 (33.9)	247 (35.9)	< 0.001

*Abbreviations: ACS* acute coronary syndrome, *AF* atrial fibrillation, *AHF* acute heart failure, *CAD* coronary artery disease, *CHF* congestive heart failure, *CPR* cardiopulmonary resuscitation, *CKD* chronic kidney disease, *COLD* chronic obstructive lung disease, *PH* pulmonary hypertension, *h* hours, *HLD* hyperlipidemia, *HTN* hypertension, *HTX* post heart transplantation, *mdn* median, *n* number, *Yr* year

**Table 4:** Thirty-day in-hospital mortality and complications of patients with prior CPR according to annualized hospital volume of VA-ECMO procedures

	30-day in-hospital mortality and complications				
	Overall (***n*** = 4429)	≤ 50 procedures/Yr (***n*** = 2637)	> 50–100 procedures/Yr (***n*** = 1104)	> 100 procedures/Yr (***n*** = 688)	***P v***alue
**30-day in-hospital mortality,*n*(%)**					
30-day in-hospital mortality	3016 (68.1)	1866 (70.8)	732 (66.3)	418 (60.8)	< 0.001
**Complications,*n*(%)**					
Bleeding	817 (18.4)	375 (14.2)	239 (21.6)	203 (29.5)	< 0.001
Stroke	40 (0.9)	17 (0.6)	15 (1.4)	8 (1.2)	0.080
Abdominal ischemia	291 (6.6)	124 (4.7)	89 (8.1)	78 (11.3)	< 0.001
Limb ischemia	335 (7.6)	160 (6.1)	106 (9.6)	69 (10.0)	< 0.001

*Abbreviations: n* number, *Yr* year

### Patient characteristics according to hospital procedure volume

In the overall cohort, the majority (53.1%, *n* = 5421) of patients was treated at hospitals in the lowest volume category (≤ 50 VA-ECMO implantations per year) in Germany. We observed significant differences in baseline characteristics of treated patients regarding the annualized procedural volume. More patients treated at hospitals in the lowest procedural volume category had ACS, prior CPR, and CAD. However, patients treated at hospitals with > 50 annual procedures had a higher percentage of AHF and CHF. There were no clinically significant differences in sex between the hospital procedure volume categories (*p* > 0.05). At hospitals with an annual procedure volume >  50 VA-ECMO implantations, we observed a longer VA-ECMO duration as compared to low-volume hospitals (Table 1).

In patients with prior CPR, there were significant differences in the patient characteristics regarding the annualized procedural volume, too. More patients with prior CPR treated at hospitals in the lowest volume category had ACS, CAD, and CKD as compared to the overall cohort. In regard to VA-ECMO duration, we found a similar trend in patients with prior CPR compared to the overall cohort (Table 3).

### Hospital procedure volume and 30-day in-hospital mortality in the overall cohort and prior CPR

**Fig. 2:**
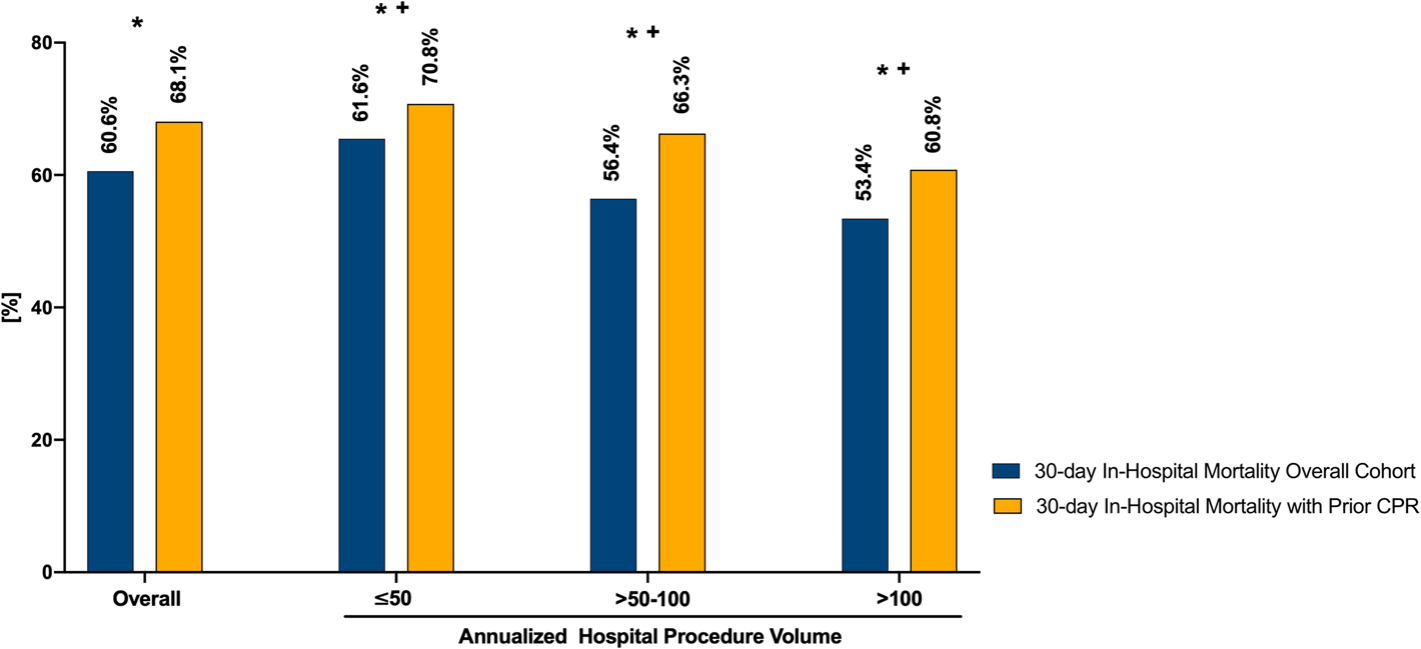
Thirty-day in-hospital mortality of the overall cohort and patients with prior CPR according to annualized hospital volume of VA-ECMO procedures. *p < 0.001: total cohort vs. prior CPR in each category (overall, ≤ 50, > 50–100, > 100). ^**+**^*p* < 0.05: ≤ 50 vs. > 50–100, ≤ 50 vs. > 100, > 50–100 vs. > 100 for the total cohort and prior CPR

The overall 30-day in-hospital mortality was 60.6% (Fig. 2, Table 2). There was a significant difference in 30-day in-hospital mortality between the groups of annualized hospital volume of VA-ECMO procedures. In detail, 30-day in-hospital mortality was significantly lower at hospitals with a higher annualized procedural volume > 50–100 or > 100 cases (30-day in-hospital mortality > 50–100 cases: 56.4%; 30-day in-hospital mortality > 100 cases: 53.4%) than at hospitals with a lower annualized procedural volume of ≤ 50 cases (30-day in-hospital mortality ≤ 50 cases: 65.0%; both *p* < 0.05).

In patients with prior CPR, 30-day in-hospital mortality was 68.1%, which was significantly higher than in the overall cohort (*p* < 0.001) (Fig. 2, Table 4). Congruently to the results of the overall cohort, 30-day in-hospital mortality was significantly lower at hospitals with a higher annualized procedural volume of > 50–100 or > 100 cases than at hospitals with a lower annualized procedural volume of ≤ 50 cases (both *p* < 0.05) in patients with prior CPR.

**Fig. 3:**
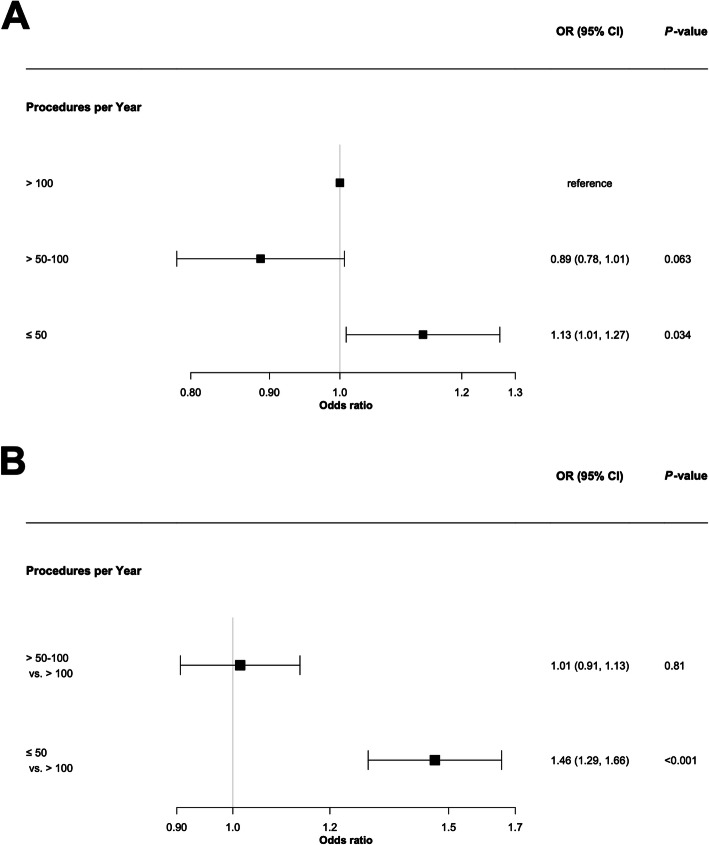
**a** Thirty-day in-hospital mortality depending on hospital procedure volume during index-hospitalization adjusted for age, sex, prior CPR, indication category, duration of VA-ECMO support, and complications (bleeding, stroke, abdominal ischemia, and limb ischemia). **b** Association between hospital procedure volume and complications (bleeding, stroke, abdominal ischemia, and limb ischemia) during index-hospitalization adjusted for age, sex, prior CPR, indication category, duration of VA-ECMO support, and time to 30-day in-hospital mortality

Multivariable adjusted logistic regression analysis showed a significant association of 30-day in-hospital mortality and hospital procedure volume. Patients treated at hospitals with ≤ 50 VA-ECMO implantations per year had a 13% higher relative risk of 30-day in-hospital mortality as compared to patients treated at hospitals with an annual procedure volume of > 100 (adjusted odds ratio 1.13, 95% confidence interval [CI] 1.01–1.27, *p* < 0.001; Fig. 3A).

### Complications according to hospital procedure volume

Among 10,207 patients undergoing VA-ECMO implantation, 2044 (20.0%) patients had major bleeding, 751 (7.4%) limb ischemia, 711 (7.0%) abdominal ischemia, and 105 (1.0%) stroke, respectively.

Adjusted multinomial logistic regression analysis revealed a higher likelihood for complications at hospitals with lower vs. higher annual procedure volume (annual procedure volume ≤ 50 vs. > 100 VA-ECMO implantations, adjusted odds ratio 1.46, 95% CI 1.29–1.66, *p* = 0.001) (Fig. 3B).

## Discussion

In this large, nationwide study, most VA-ECMO cases were treated at hospitals with a lower annual procedure volume. Treatment at a hospital with a lower annual procedure volume was associated with a higher mortality risk and likelihood for complications compared to higher volume centers.

Previous studies have shown that higher procedural volumes of cardiovascular interventions were associated with lower mortality rates [6, 8, 9]. Selected medical conditions including VA-ECMO in cardiac failure and critical care conditions such as mechanical ventilation are shown to have a significant volume-outcome association in which higher hospital case numbers are associated with survival benefit [15–17]. Particularly, an international study using hospital ECMO volumes from 290 international centers found strong associations of higher hospital-level ECMO volume and lower mortality for neonates and adults, but not for children [18]. A similar observation has been made in Japan; for cases involving ECMO for respiratory failure, a higher hospital procedure volume of ECMO treatment for any indications was associated with lower in-hospital mortality [19]. Contrary to the traditional volume-outcome relationship, other studies on VA-ECMO reported increased mortality associated with high-volume hospitals and in patients transferred to tertiary centers [20, 21]. However, additional information is needed about patient characteristics and selection, management strategies for VA-ECMO, and organization of care within high- and low-volume centers to improve treatment and outcomes among these critically ill patients. In the present study, the majority of patients with VA-ECMO support were treated at hospitals with a low procedural volume (≤ 50 VA-ECMO implantations per year). However, 30-day in-hospital mortality across the categories of hospital procedural volume showed a significant difference between the lowest and highest annualized hospital procedural volumes. Lowest 30-day in-hospital mortality was observed at hospitals with an annualized hospital procedural volume > 100 VA-ECMO cases, especially. However, at hospitals in the lowest procedural volume category, a greater proportion of patients with ACS, prior CPR, CAD, CKD, and advanced age underwent therapy. In contrast, patients treated in the highest procedural volume categories showed a higher proportion of AHF and CHF, whereas sex differences between the hospital procedure volume categories were not observed. As expected across all categorical divisions and compared to the overall cohort, patients with prior CPR had a higher 30-day in-hospital mortality on VA-ECMO in general. Again, differences in baseline criteria were observed between the groups. The proportion of patients suffering from ACS decreased with a higher procedural volume. At hospitals with ≥ 50 annual procedural volume, the leading indication for VA-ECMO implantation slightly shifts from ACS to AHF. Significant differences in patient characteristics indicated by a higher proportion of patients with ACS, prior CPR, and comorbidities such as CAD and CKD in lowest procedural volume category could explain the higher 30-day in-hospital mortality compared to the highest volume categories with > 50 VA-ECMO implantations per year. However, after adjusting for potential confounders, the significant association with an increased risk in patients treated at lower volume centers persisted.

VA-ECMO is a potentially life-saving therapy for reversible circulatory failure not responsive to conventional therapies [3, 22]. However, complications on VA-ECMO are very common and are associated with a significant increase in morbidity and mortality [4, 23].

In the present study, we observed high complication rates of bleeding with 20.0%, limb ischemia with 7.4%, abdominal ischemia with 7.0%, and stroke with 1.0% in the overall cohort.

Stroke occurs in approximately 4% of VA-ECMO patients [11, 24]. This is similar to our results. In addition, the rate of stroke varies by indication and cannulation technique. It has been reported that femoral artery cannulation had a noticeably lower risk compared to central access [25]. Furthermore, the cause of stroke is multifactorial and can be associated with hemodynamic instability and thromboembolic events [26].

In a meta-analysis of 1866 patients by Cheng et al., lower extremity ischemia occurred in 16.9% of patients undergoing VA-ECMO support [11]. However, our analysis showed a lower complication rate. Although this might be explained by underreporting in the used registry, the use of prophylactic antegrade perfusion catheters might have reduced the frequency of limb ischemia over time [27].

Abdominal ischemia during VA-ECMO support is a known complication. To date, incidence rate of bowel and intestinal ischemia on VA-ECMO therapy is largely unknown. Among the total cohort, the incidence rate of abdominal ischemia was 6.7%. Hence, it can be deduced that abdominal ischemia is a relevant complication on VA-ECMO therapy and should be carefully considered before initiation of this invasive treatment.

We found a strong association between hospital procedure volume and complications. Although the crude incidence of complications was higher in hospital with a higher procedural volume, after adjusting for confounders, a higher likelihood for complications was observed at hospitals with lower vs. higher annual VA-ECMO volume. This finding is in line with previous cardiovascular and cardiac surgical studies that have demonstrated a relationship between higher hospital procedure or operator volume and fewer complication rates [28–30].

The observation that higher procedural experience for VA-ECMO was associated with better in-hospital outcomes could be explained by differences in patient characteristics, technical factors, and more experienced handling of patients with complications at higher volume centers.

### Limitations

This is a retrospective study based on administrative data, with its inherent limitations. Individual additional follow-up data with long-term survival and complication rates were not available. Therefore, analysis of predisposing factors for ischemic and bleeding complications might be biased. As known from other registries, incidence of complication rates is potentially misreported. Moreover, medical approaches applied prior to VA-ECMO implementation were not available (e.g., timing of inotrope and vasopressor initiation and dosing, etc.). In this regard, patient complexity and severity of illness were not adjusted for in the current model. We did not have data on complications after hospital discharge which might have impacted our results.

Moreover, differences in institutional structure, processes, and thresholds to initiate VA-ECMO therapy potentially bias outcomes and must be considered when interpreting this study. The exact time course of the different diagnoses as being prevalent at admission or incident during the hospital stay is not possible in the current data set. Finally, our data is limited to data for Germany and may not be generalizable to other health care systems.

## Conclusions

In a large nationwide registry with more than 10,000 patients, a significant volume-mortality association was shown for VA-ECMO procedures in cardiogenic shock. The majority of patients with VA-ECMO support were treated at hospitals with a low procedural volume in Germany. Thirty-day in-hospital mortality risk and likelihood of complications were higher at hospitals with the lowest annual procedure volume.

## Supplementary information

**Additional file 1.**

## Data Availability

Data and material are available.
