# Supplementary material for: Procedural volume and outcomes in patients undergoing VA-ECMO support
Source: Crit Care. 2020 Jun 5;24:291. doi: 10.1186/s13054-020-03016-z (PMC7275456; doi:10.1186/s13054-020-03016-z)
Supplement: Supplementary file 1 — Additional file 1. [file 13054_2020_3016_MOESM1_ESM.docx]

**Supplemental Material**

This Supplement Material formed part of the original submission and has been peer reviewed. We post it as supplied by the authors.

Supplemental Material: Becher et al., Procedural Volume and Outcomes in Patients undergoing VA-ECMO.

**Supplemental Material**

Supplemental Material: Becher et al., Procedural Volume and Outcomes in Patients undergoing VA-ECMO.

**Table of Contents**  Page number

Supplemental Tables

Table I. Baseline characteristics, diagnosis and procedure codes. 3

Table II. Indication categories, diagnosis and procedure codes. 5

Table III. Complications, diagnosis and procedure codes. 11

**Supplemental Tables**

**Table I.** Baseline characteristics, diagnosis and procedure codes.

| **Baseline characteristics** | **International Classification of Diseases, 10th Edition, Clinical Modification codes** |
| --- | --- |
| Atrial fibrillation | Diagnosis codes (from index and prior admissions)  I4891, I4892 |
| Hypertension | Diagnosis codes (from index and prior admissions)  I150, I1500, I1501, I151, I1511, I152, I1520, I1521, I158, I1580, I1581, I159, I1590, I1591, I100, I1001, I101, I1011, I109, I1091, I11, I110, I1101, I1190, I1191 |
| Hyperlipidemia | Diagnosis codes (from index and prior admissions)  E78.x |
| Pulmonary hypertension | Diagnosis codes (from index and prior admissions)  I27.x |
| Diabetes | E10.x, E11.x, E14.x |
| Coronary artery disease | Diagnosis codes (from index and prior admissions)  I2109, I2119, I2111, I2129, I214, I213, I241, I200, I240, I248, I252, I208, I201, I209, I2510, I25810, I25811, I25812, I253, I2541, I2542, I2582, I2583, I2584, I255, I2489, I259, I511, I512, I510, I230  Procedure codes (from index and prior admissions)  8837.x, 5361.x, 5362.x, 5363.x |
| Congestive heart failure | Diagnosis codes (from index and prior admissions)  I0981, I509, I501, I5020, I502, I5022, I5023, I5030, I5031, I5032, I5033, I5040, I5041, I5042, I5043, I509, I514, I515, I2510, I517, I970, I97110, I97130, I97190, I511, I512, I510, I230, I5189, I5181, I519 |
| Chronic obstructive lung disease | Diagnosis codes (from index and prior admissions)  J410, J411, J449, J441, J440, J418, J42, J439, J4520, J4522, J4521, J4520, J4522, J449, J440, J441, J45990, J45991, J45909, J45998, J45902, J45901, J479, J471, J449 |
| Chronic kidney disease | Diagnosis codes (from index and prior admissions)  I120, I129, I1310, I130, I1311, I132, N032, N033, N035, N038, N08, N038, N039, N059, N052, N055, N171, N172, N08, N058, N059, N181, N182, N183, N184, N185, N186, N189, N19, N269, N250, N251, N2581, N2589, N259, Z940, Z4931, Z4901, Z4902, Z4931, Z4932 |
| Sepsis | Diagnosis codes (from index and prior admissions)  A409, A412, A4101, A4102, A411, A403, A414, A4150, A413, A4151, A4152, A4153, A4159, A4189, A419, B955, B950, B951, B954, B952, B958, B9561, B9562, B957, B953, B961,B9621, B9622, B9623, B9620, B9629, B963, B964, B965, A493, B960, B966, B967, B9689, B968, A880, B59, J120, J121, J122, J1281, J1289, J129, J13, J181, J150, J151, J14, J154, J153, J1520, J15211, J15212, J1529, J158, J155, A481, J158, J159, J157, J160, J168, B250, A3791, A221, B440, J17, J180, J189, J1100, J129, J111, J112, J1181, J1189, J690, J691, J698, J860, J869, J850, J851, J852, J853, P249, P2400, P2401, P2410, P2411, P2420, P2421, P2480, P2481, K352, K353, K3580, K3589, K37, K36, K5712, K5713, K5732, K5733, K610, K611, K613, K67, K658, K650, K651, K652, K6812, K6819, K689, K653, K654, K658, K659, K630, K750, K751, K810, K819, N110, N118, N10, N151 N2884, N2885, N2886, N12, N16, N159, N410, N411, N412, N413, N51, N414, N418, N419, N7001, N7002, N7003, N7011, N7012, N7013, N7091, N7092, N7093, N730, N731, N732, N733, N736, N734, N738, N739, N710, N711, N719, N72, N760, N761, N762, N763, N771, N750, N751, N764, N766, N770, N7681, N759, N765, N7689, N739, L03019, L03029, L03039, L03049, L049, L0100, L0103, L0501, L0502, L0591, L0592, L080, L88, L0889, L980, E832, L0889, L089, P369, P393, R572, R7881, P393, P398, R7881, A419, R6520 |
| Liver disease | Diagnosis codes (from index and prior admissions)  B150, B159, B162, B1911, B160, B181, B169, B1910, B161, B181, B180, B1711, B170, B172, B182, B178, B1710, B188, B189, B190, B1920, B1921, B199, I8501, I8500, I8511, I8510, I860, I861, I862, I863, I868, K7200, K700, K7010, K7030, K709, K739, K730, K754, K732, K738, K740, K7460, K7469, K743, K744, K745, K760, K7689, K741, K769, K750, K751, K7290, K7291, K766, K767, K7210, K7290, K761, K77, K716, K759, K763, K7581, K7689, K769, R17, R160, R180, R188, R740, R748, R945, Z944 |
| Cardiopulmonary resuscitation | Procedure codes (from index and prior admissions)  8771 |

**Table II.** Indication categories, diagnosis and procedure codes.

| **Indication categories** | **International Classification of Diseases, 10th Edition, Clinical Modification codes** |
| --- | --- |
| Complications post heart transplant | Heart transplantation  Procedure codes (ECMO placed on the day of or after heart transplantation)  3751 |
| Acute coronary syndrome | ST elevated myocardial infarction  Diagnosis codes (primary diagnosis or present on admission)  I2109, I2119, I211, I2119, I2129  Non-ST elevated myocardial infarction  Diagnosis codes (primary diagnosis or present on admission)  I214, I213  Percutaneous coronary intervention  Procedure codes (ECMO placed anytime relative to procedure)  8837.x  Coronary artery bypass graft  Procedure codes (ECMO placed prior to procedure)  5361.x, 5362.x, 5363.x |
| Cardiac failure following procedure | Valvuloplasty or valvotomy  Procedure codes (ECMO placed on day of or after procedure)  5350.x, 5353.x  Valve replacement  Procedure codes (ECMO placed on day of or after procedure)  5351.x, 5352.x  Other valve operations  Procedure codes (ECMO placed on day of or after procedure)  535a.x  Operation structures adjacent to heart valves  Procedure codes (ECMO placed on day of or after procedure)  5354.x  Septal operation  Procedure codes (ECMO placed on day of or after procedure)  5355.x, 5356.x  Coronary artery bypass graft  Procedure codes (ECMO placed on day of or after procedure)  5361.x, 5362.x, 5363.x  Cardiotomy and pericardiotomy  Procedure codes (ECMO placed on day of or after procedure)  5372.x, 5373.x, 8835.x, 5379.1  Other repair of heart and pericardium  Procedure codes (ECMO placed on day of or after procedure)  5374.x  Pacemaker procedures  Procedure codes (ECMO placed on day of or after procedure)  5377.x, 5378.x, 5379.x, 537a  Other non-cardiac procedures  Procedure codes (ECMO placed on day of or after procedure)  538.x, 539.x, 54.x, 501-599.x |
| Acute heart failure | Rheumatic valve disease  Diagnosis code (primary diagnosis or present on admission)  I050, I051, I058, I060, I061, I062, I068, I069, I080, I088, I089, I071, I072, I078  Non-rheumatic valve disease  Diagnosis code (primary diagnosis or present on admission)  I340, I348, I350, I351, I352, I358, I359, I360, I368, I370, I378  Myocarditis  Diagnosis code (primary diagnosis or present on admission)  I41, I409, I400, I401, I408, I012, B3322, A3681  Pericarditis  Diagnosis code (primary diagnosis or present on admission)  I32, I010, A3953, B3323  Cardiomyopathy  Diagnosis code (primary diagnosis or present on admission)  I423, I42, I422, I428, I424, I425, I428, I426, I43, I427  Conduction disorders  Diagnosis code (primary diagnosis or present on admission)  I442, I4430, I440, I441, I444, I445, I4460, I4469, I447, I4510, I4430, I4439, I454, I452, I453, I455, I456, I4581, I4589, I459  Cardiac dysrhythmia  Diagnosis code (primary diagnosis or present on admission)  I471, I472, I479, I4891, I4892, I4901, I4902, I469, I4940, I491, I493, I4949, I495, I498, I499, R001  Acute heart failure  Diagnosis code (primary diagnosis or present on admission)  I5020, I5021, I5023, I5030, I5033, I5040, I5041, I5043, I501  Shock  Diagnosis code (primary diagnosis or present on admission)  R579, R570, R6521, R571, R578  Aneurysm and dissection of heart  Diagnosis code (primary diagnosis or present on admission)  I253, I2541, I2542, I253  Aortic dissection  Diagnosis code (primary diagnosis or present on admission)  I7100, I7101, I7102, I7103  Ruptured aortic aneurysm  Diagnosis code (primary diagnosis or present on admission)  I711, I713, I718, I715  Hemothorax  Diagnosis code (primary diagnosis or present on admission)  S271XXA, S21309A, S271XXA  Pneumohemothorax  Diagnosis code (primary diagnosis or present on admission)  S272XXA, S21309A, S272XXA  Cardiac injury  Diagnosis code (primary diagnosis or present on admission)  S2610XA, S2690XA, S2699XA, S2691XA, S2692XA, S2692XA  Iatrogenic cardiac complications  Diagnosis code (primary diagnosis or present on admission)  T82519A, T82529A, T82539A, T82599A, T82110A, T82111A, T82120A, T82121A, T82190A, T82191A, T8201XA, T8202XA, T8203XA, T8209XA, T82211A, T82212A, T82213A, T82218A, T82110A, T82111A, T82120A, T82121A, T82190A, T82191A, T82221A, T82222A, T82223A, T82228A, T82518A, T82528A, T82538A, T82598A, T82390A, T82391A, T82392A, T8249XA, T82590A, T82591A, T82593A, T82595A, T82598A, T826XXA, T827XXA, T827XXA, T82817A, T82827A, T82837A, T82847A, T82857A, T82867A, T82897A, T829XXA, T82817A, T82827A, T82837A, T82847A, T82857A, T82867A, T82897A, T829XXA, T82818A, T82828A, T82838A, T82848A, T82858A, T82868A, T82898A, T829XXA, T8620, T8621, T8622, I97710, I97790, I9788, I9789 |
| Respiratory failure | Acute respiratory failure  Diagnosis code (primary diagnosis or present on admission)  J9600, J9690, J80, J9620, R092  Pulmonary embolism  Diagnosis code (primary diagnosis or present on admission)  I2690, I2699, T800XXA, T81718A, T8172XA  T82817A, T82818A, I2690, I2692, I2699  Pulmonary congestion  Diagnosis code (primary diagnosis or present on admission)  J182, J811  Acute pulmonary edema  Diagnosis code (primary diagnosis or present on admission)  J810  Pulmonary collapse  Diagnosis code (primary diagnosis or present on admission)  J930, J95811, J9311, J9312, J9381, J9382, J9383, J939, J9811  J9819  Pneumonia  Diagnosis code (primary diagnosis or present on admission)  A0222, A202, A212, A221, A310, A420, B012, B052, A70, J17, A78, B371, B380, B381, B382, B392, B395, J17, B399, B583, B59, J120, J121, J122, J1281, J1289, J129, J13, J181, J150, J151, J14, J153, J154, J1520, J15211, J15212, J1529, J158, J155, J156, A481, J159, J157, J160, J168, B250, A3791, A221, B440, J180, J189, J850, J851, J852, J17  Influenza  Diagnosis code (primary diagnosis or present on admission)  J1100, J129, J111, J112, J1181, J1189, J09X1, J09X2, J09X3, J09X9  Other lower respiratory infections  Diagnosis code (primary diagnosis or present on admission)  J209, J210, J218, J860, J869, J869, J941, J948, J949, R091, J90, J942, J910, J90, J942, J948, J918, J850, J851, J852, J853  Acute bronchitis  Diagnosis code (primary diagnosis or present on admission)  J40, J411, J441, J440  Emphysema  Diagnosis code (primary diagnosis or present on admission)  J439  Acute bronchiectasis  Diagnosis code (primary diagnosis or present on admission)  J471, J441  Cystic fibrosis  Diagnosis code (primary diagnosis or present on admission)  E849, E8411, E840, E8419, E848  Pneumoconioses  Diagnosis code (primary diagnosis or present on admission)  J60, J61, J628, J63.x, J660, J661, J662, J668, J64  Other environmental exposures  Diagnosis code (primary diagnosis or present on admission)  J670, J671, J672, J673, J674, J675, J676, J677, J678, J679, J680, J681, J682, J683, J690, J691, J698, J700, J701, J705, J708, J709  Pulmonary fibrosis  Diagnosis code (primary diagnosis or present on admission)  J8410, J8489, J84111, J84112, J84113, J84114, J84115, J842, J84116, J84117  Bronchus and lung malignancies  Diagnosis code (primary diagnosis or present on admission)  D869, C33, C3400, C3410, C342, C3430, C3480, C3490  Iatrogenic lung injury  Diagnosis code (primary diagnosis or present on admission)  J95821, J9600, J951, J952, J953, J95822, J9620, T86810, T86811, T86819  Other acute respiratory conditions  Diagnosis code (primary diagnosis or present on admission)  J8401, J8403, J8402, J84111, J84112, J84113, J84114, J84115, J842, J84116, J84117, J8481, J8482, J84841, J84842, J8483, J84843, J84848, J8409, J849, J17, M3481, J99, J982, J983, J82, B4481, J9584, J9600, J9690, J80, J9610, J9620, J984 |

**Table III.** Complications, diagnosis and procedure codes.

| **Complications** | **International Classification of Diseases, 10th Edition, Clinical Modification codes** |
| --- | --- |
| Stroke | Iatrogenic stroke  Diagnosis code (from index hospitalization or primary diagnosis of  subsequent admissions)  I97811, I97821  Hemorrhagic stroke  Diagnosis codes (from index hospitalization or primary diagnosis of  subsequent admissions)  I609, I619, I621, I6200, I629  Ischemic stroke  Diagnosis codes (from index hospitalization or primary diagnosis of  subsequent admissions)  I6322, I63139, I63239, I63019, I63119, I63219, I6359, I6320, I6330, I6340, I6350 |
| Bleeding | Post-operative hemorrhage  Diagnosis codes (from index hospitalization or primary diagnosis of  subsequent admissions)  D7801, D7802, D7821, D7822, E3601, E3602, G9731, G9732, G9751, G9752, H59111, H59112, H59113, H59119, H59121, H59123, H59129, H59311, H59312, H59313, H59319, H59321, H59322, H59323, H59329, H9521, H9522, H9541, H9542, I97410, I97411, I97418, I9742, I97610, I97611, I97618, I9762, J9561, J9562, J95830, J95831, K9161, K9162, K91840, K91841, L7601, L7602, L7621, L7622, M96810, M96811, M96830, M96831, N9961, N99820, N99821  Intracerebral hemorrhage  Diagnosis codes (from index hospitalization or primary diagnosis of  subsequent admissions)  I609, I619, I621, I6200, I629  Hemopericardium or cardiac tamponade  Diagnosis codes (from index hospitalization or primary diagnosis of  subsequent admissions)  I312, I314  Gastrointestinal hemorrhage (acute and chronic)  Diagnosis codes (from index hospitalization or primary diagnosis of  subsequent admissions)  I8501, I8511, R58, K2211, K250, K252, K260, K262, K270, K272, K280, K282, K625, K920, K921, K922, K254, K256, K264, K266, K270, K274, K276, K284, K286, K2901, K2941, K2951, K2961, K2921, K2961, K2971, K2991, K2981, K5281  Hematuria  Diagnosis codes (from index hospitalization or primary diagnosis of  subsequent admissions)  R319, R310, R311, R312  Hemarthrosis  Diagnosis codes (from index hospitalization or primary diagnosis of  subsequent admissions)  M2500, M25019, M25029, M25039, M25049, M25059, M25069, M25073, M25076, M2508, M2500  Hemoptysis  Diagnosis codes (from index hospitalization or primary diagnosis of  subsequent admissions)  R041, R042, R049, R0481, R0489  Epistaxis  Diagnosis codes (from index hospitalization or primary diagnosis of  subsequent admissions)  R040  Retinal/choroidal hemorrhage  Diagnosis codes (from index hospitalization or primary diagnosis of  subsequent admissions)  H3560, H35739, H31309, H31319, H31329 |
| Abdominal ischemia | Abdominal ischemia  Diagnosis codes (from index hospitalization or primary diagnosis of subsequent admissions)  K55, R1980, R10, R190  Procedure codes (from index hospitalization or primary diagnosis of  subsequent admissions)  55410 |
| Limb ischemia | Limb ischemia  Diagnosis codes (from index hospitalization or primary diagnosis of  subsequent admissions)  I739, I771, T796, M622  Procedure codes (from index hospitalization or primary diagnosis of  subsequent admissions)  5851 |
